# Modeling Motorcyclists’ Aggressive Driving Behavior Using Computational and Statistical Analysis of Real-Time Driving Data to Improve Road Safety and Reduce Accidents

**DOI:** 10.3390/ijerph19137704

**Published:** 2022-06-23

**Authors:** Sarah Najm Abdulwahid, Moamin A. Mahmoud, Nazrita Ibrahim, Bilal Bahaa Zaidan, Hussein Ali Ameen

**Affiliations:** 1 College of Graduate Studies, Universiti Tenaga Nasional, Kajang 43000, Malaysia; 2 Institute of Informatics and Computing in Energy, Department of Computing, College of Computing and Informatics, Universiti Tenaga Nasional, Kajang 43000, Malaysia; nazrita@uniten.edu.my; 3 Future Technology Research Center, National Yunlin University of Science and Technology, Douliu 64002, Taiwan; zaidanbb@yuntech.edu.tw; 4 Department of Computer Techniques Engineering, Al-Mustaqbal University College, Hillah 51001, Iraq; hussein_awadh@mustaqbal-college.edu.iq

**Keywords:** aggressive behavior modeling, real-time data analysis, traffic violation, motorcyclists, road safety

## Abstract

Driving behavior is considered one of the most important factors in all road crashes, accounting for 40% of all fatal and serious accidents. Moreover, aggressive driving is the leading cause of traffic accidents that jeopardize human life and property. By evaluating data collected by various collection devices, it is possible to detect dangerous and aggressive driving, which is a huge step toward altering the situation. The utilization of driving data, which has arisen as a new tool for assessing the style of driving, has lately moved the concentration of aggressive recognition research. The goal of this study is to detect dangerous and aggressive driving profiles utilizing data gathered from motorcyclists and smartphone APPs that run on the Android operating system. A two-stage method is used: first, determine driver profile thresholds (rules), then differentiate between non-aggressive and aggressive driving and show the harmful conduct for producing the needed outcome. The data were collected from motorcycles using -Speedometer GPS-, an application based on the Android system, supplemented with spatiotemporal information. After the completion of data collection, preprocessing of the raw data was conducted to make them ready for use. The next steps were extracting the relevant features and developing the classification model, which consists of the transformation of patterns into features that are considered a compressed representation. Lastly, this study discovered a collection of key characteristics which might be used to categorize driving behavior as aggressive, normal, or dangerous. The results also revealed major safety issues related to driving behavior while riding a motorcycle, providing valuable insight into improving road safety and reducing accidents.

## 1. Introduction

Every year, the number of people who lose their lives on the road increases [1,2]. Since the year 2003, the rate of motorbike fatalities has been the main factor reason for the constant increase in of crash deaths [3]. Driving behavior factors cause over 95% of these incidents [4,5]. Malaysia is ranked 19th out of 182 nations concerning the highest number of road traffic fatalities for every 100,000 inhabitants [6]. Furthermore, Malaysia has Asia’s second-highest mortality rate [1], moreover, it is recently rated fifth in the world among nations; however various government initiatives aimed at decreasing road accidents, there is no evidence of a global decline in traffic fatalities. If preventive actions are not taken immediately, these road accidents will become the fifth-greatest cause of mortality all over the world by 2030 [2].

Driving behavior is the leading cause of traffic fatalities [7,8]. Therefore, preventing aggressive or dangerous driving attitudes is a critical first step toward taking action for decreasing them. For improving the safety of roads, it is important to evaluate an individual’s driving style and identify aggressive conduct. Recommendations must be made for guiding drivers toward normal driving behavior [9,10]. In addition, aggressive driving is considered the driver’s behavior categorization or pattern that includes normal speeding and inconsistent or excessive acceleration and deceleration, which is a significant cause in more than 90% of road crashes. For gaining a better knowledge of driving behavior, researchers explored a variety of methodologies [11]. However, as previous research has shown, techniques based on self-reported behavior, demographic profiles [12], and personal risk perceptions tend to presume that behavior in groups is homogeneous. Furthermore, user viewpoint is subject to questionnaires and self-reports [13,14,15,16,17], which may create a mistaken sense of insecure/secure behavior. Moreover, sensitivity to aggressive driving behavior might be proven by raising average speed by 5%, which results in a 20% and 10% increase in fatal crashes and injury-causing, respectively [6]. The following research questions emerge from the literature review:

1. What is the driving style of Malaysian drivers that suggests or conforms to aggressive behavior?
2. What is the average of normal, aggressive, and dangerous driving for Malaysian drivers?

Driver safety cannot be ensured solely by monitoring driving behavior. Achieving effective and normal driving requires recognizing aggressive driving to encourage more effective traffic safety studies [13,18]. We employ an approach for modeling drivers’ speeds in this study to quantify and analyze individual driving behavior with the use of recorded data from the -Speedometer GPS-, an application based on the Android system, supplemented with spatiotemporal information [9]. The following are some of the study’s important contributions:

1. Indicating the needed normal behaviors via direct observation.
2. Identifying behavior and quantifying normal/aggressive and dangerous behavior.
3. Assessing and monitoring changes in normal behavior and performance, and providing feedback.

Driving behavior identification is a prerequisite for traffic research, and it yields advantages in two areas particularly: better understanding of driving behavior and improving road safety. Driving behavior research is extremely useful for a variety of objectives in transportation engineering. Objectives include data collection for statistical analysis, and identifying driving models and parameter estimates. Experiments using motorcycles and a mobile application (AndroSensor V1.9.6.3, Speedometer GPS version: 3.7.76) were used for collecting data about driving behavior. The current study is organized as follows: First, the study’s literature review is presented. Second, the approach and data acquired are indicated. Third, the data analyses are explained, and findings are given. Lastly, findings are explored, with suggestions for further research.

## 2. Related Studies

Many researchers have used technological advancements to acquire richer and more specific data to increase our understanding of driving behavior. As a result, driving behavior records have grown significantly larger and more complicated, demanding new approaches for analysis, such as driving behavior profiles. In studies of driving behavior, GPS devices and other in-vehicle sensors have decreased the number of challenges connected with traditional kinds of data collection [19,20]. This includes a proclivity for over-or under-reporting driving behavior (particularly speeding) and limited time-series data contained in enforcement records and self-reported surveys [2].

Accelerometers [21,22,23], on-board diagnostics (OBD) [24], GPS sensing [25,26], gyroscopes [24,25], and ProLaser-III with motorcycles [27] have all been used in research studies related to road accidents. Despite the many disadvantages of the devices, they at least offer a complete record regarding day-to-day driving [11]. Even though the quantity of real-time driving studies (as research using this technology is identified) is restricted because of the resources and expenses required, they are becoming more frequent [28]. In human factor studies and intelligent transportation systems, driving behavior analysis is prevalent. Many studies have attempted to comprehend driving behavior [9]. The utilization of available sensors to offer information linked to the driver’s status and behavior is one of the main themes for driving behavior analysis. Utilizing external data gathering devices which supply additional information to the system provides driver monitoring [29].

Driving behavior is characterized in the literature as a driver’s various manners, habits, and acts while driving, which are grouped into three styles: aggressive driving, normal or safe driving, and dangerous driving [30,31]. Normal driving behavior is defined by a few studies as a driver’s typical day-to-day practices. Abnormal driving behavior, on the other hand, is characterized as a driver’s uncommon behavior when influenced by mental or physical factors [32]. This behavior was viewed as a one-class classification problem [33]. Since drivers may be hooked to abnormal driving behavior and the majority of their driving behaviors contain anomalous behaviors, such a definition for driving behavior is inadequate. The features of driving behavior were researched to differentiate distinct driving behavior types, and the results are given here.

### 2.1. Normal Driving Style

Normal driving behavior, also called “typical” or “safe” driving, refers to a driver’s behavior when the driver avoids unsafe activities and reactions [11]. Normal driving style is defined as driving with no dangerous behaviors and without the qualities listed in aggressive driving, careless driving, alcoholic driving, and drowsy driving [34]. Another study defines a secure driving style as one in which the driver correctly focuses on driving [29]. Rapid changes in acceleration or speed, tailgating, dangerous lane changes, improper vehicle lateral position, driving when drunk or tired, and inattention to the driving activity must all be avoided in a normal driving style.

### 2.2. Aggressive Driving Style

The aggressive usage of a motor vehicle is likely to endanger life by raising the probability of a collision. Hostility, annoyance, impatience, or a desire to save time are common motivators for this conduct. Abrupt and anomalous changes in the speed of the vehicle, tailgating, dangerous lane changes, improper vehicle lateral position, and quick deceleration and acceleration take-off or braking are all examples of dangerous driving styles [3,35]. Aggressive driving style is also associated with driving excessively fast, recklessly overtaking or driving aggressively, infrequent use of brakes, vehicle wobbling on the road, delayed acceleration or gear change, and exceeding speed limits [34].

Visual-feature-based driving behavior identification approaches are very sensitive to light conditions. Despite the use of contemporary cameras, significant changes in light intensity reduce the precision of such procedures. Intrusive technologies or cameras may upset the driver. Image processing necessitates a large amount of computer power, making it unsuitable for real-time applications and embedded systems in automobiles. Driving behavior recognition systems depending on non-visual features, on the other hand, require little processing resources, yet have low accuracy. As a result, we propose a strategy depending on the driver’s non-visual characteristics, which provides great efficiency and accuracy.

### 2.3. Dangerous Driving Style

Dangerous driving can be defined as deliberate deviations from safe driving [36]. It includes a wide range of on-road violations such as speeding and dangerous overtaking, among others. As all these behaviors are linked with accident involvement and can cause injuries to the driver, passengers, and other people on the road, as well as economic, property, and road infrastructure damages, they deserve attention from a traffic safety perspective [37]. Dangerous driving includes a broad variety of behaviors that are often identified as aggressive driving, thus emphasizing the need to establish distinct definitions of aggressive driving and dangerous driving [36,38].

## 3. Methods

A field experiment has been carried out on the road via different paths at Universiti Tenaga Nasional (UNITEN) and Universiti Pendidikan Sultan Idris (UPSI), Malaysia. The stretch has a total length of 10 km with an average riding time of 20 min. It should be noted here that these study sites were selected due to their accessibility by the participants in the experiment during the restriction period instigated by the COVID-19 condition. Moreover, data for driving behavior were continuously recorded in every session.

### 3.1. Study Sites and Participants

**Figure 1:**
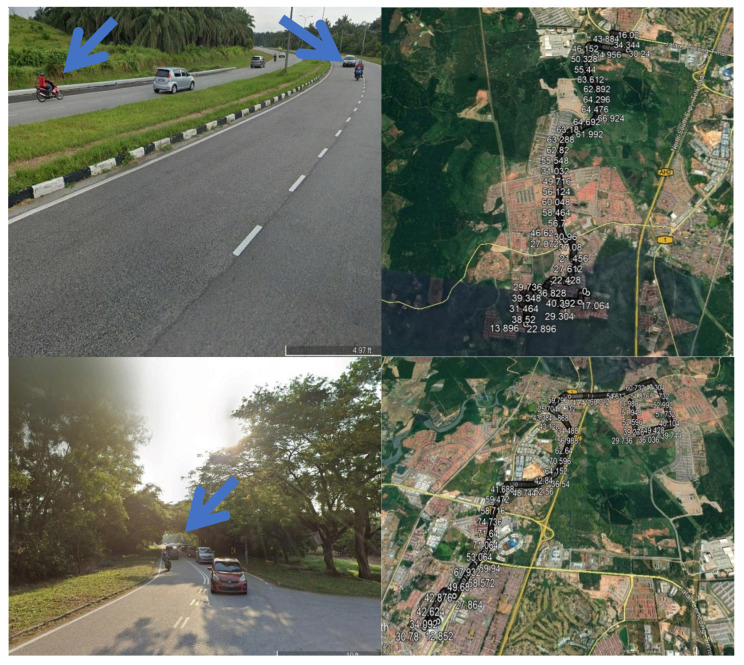
Examples of the driving path on Google Maps.

Throughout the data collection phase of this study, a total of 16 drivers participated in various motorcycle and normal driving scenarios for collecting driving data. Figure 1 illustrates road plan samples from Google Maps with several research sites. The target participants are local Malaysian students, between the ages of 18 and 35 from UPSI and UNITEN, who are familiar with the different driving routes in the city and on the highway in the area of Tanjung Malim and Selangor.

Each driver drove around 60.5 km for about 120 min on this route for a minimum of three trips. Every trip takes at least 20 min and covers at least 10 km. An instruction was sent to every driver via the WhatsApp application to ensure all drivers were following the same route. Each driver was compensated with RM15.00 (Fifteen Malaysian Ringgit) for every trip.

### 3.2. Driving Behavior Data Collection

Data collection can be defined as the process of acquiring and evaluating the information on variables of interest in a systematic manner that allows one to answer research questions, assess results, and test hypotheses. The information provided in this study consists of quantitative data, which are specified as the value of data expressed in numbers or counts, with each one of the datasets having a distinct numerical value. In addition, these data consist of any quantifiable information that may be utilized for statistical analysis and mathematical computations of real-world decisions depending on the results. A systematic method of investigation where numerical data are acquired and/or the researcher translates what is observed or collected into numerical data is known as quantitative data analysis.

The data collected in this study were based on real-world experimental testing by using a two-road driving scenario (urban (in-city) and highway) to capture driving behavior. Special devices are used to record data when driving, which consists of driving speed, acceleration, and deceleration by the drivers. The data collected must be filtered for creating an adequate research database. On the other hand, the database’s primary goal is to capture real-time driving behavior on site.

#### 3.2.1. Speed Data Collection for Driving Behavior

**Figure 2:**
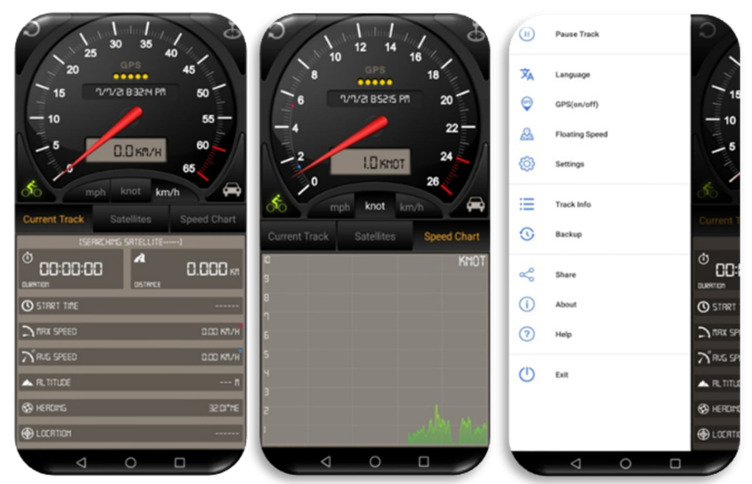
Home page of Speedometer GPS, version 3.7.76.

Data were collected from the Speedometer GPS (version 3.7.76) smartphone application for Android devices. Figure 2 shows the home page of the app.

**Figure 3:**
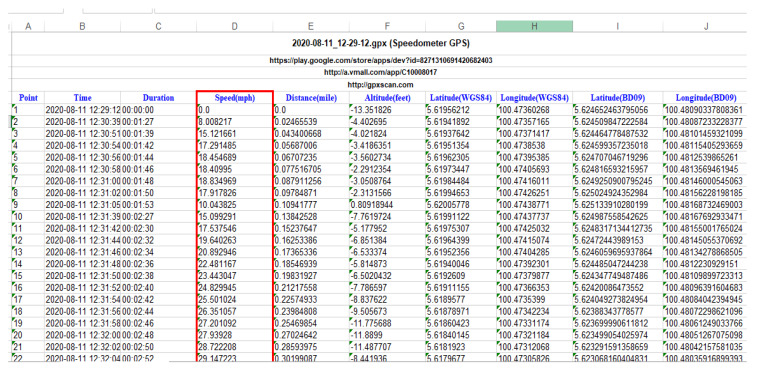
Data recorded with Speedometer GPS application.

The application runs in the background of the smartphone’s OS, requiring no user interaction when commuting. Duration, time, distance (miles), speed (mph), latitude (WGS84), longitude (WGS84), altitude (feet), latitude (BD09), and longitude (BD09) are some of the criteria used by the application to capture raw data from a smartphone (Figure 3). The speed attribute is used to indicate if the speed is over the speed limit or not; the motorcycle speed is checked with the latitude and longitude. Kilometers per hour (km/h) are used for calculating speed. The information was stored as an Excel sheet and then uploaded to a storage location for further processing. After that, data noise was removed from the database. As a result, the database provided for this experiment included 116 trips taken by 16 different drivers from June to December 2020. Lastly, speed variables regarding each one of the trips were extracted and used as input to RapidMiner Studio to detect trip events, where RapidMiner’s data science platform is intended to support many analytics users across a broad AI (Artificial Intelligent) lifecycle. The speed is calculated by kilometers per hour (km/h). The speed attribute was used as a factor to analyze driving behavior to indicate if the speed was over the speed limit or not.

#### 3.2.2. Acceleration and Deceleration (Braking)

**Figure 4:**
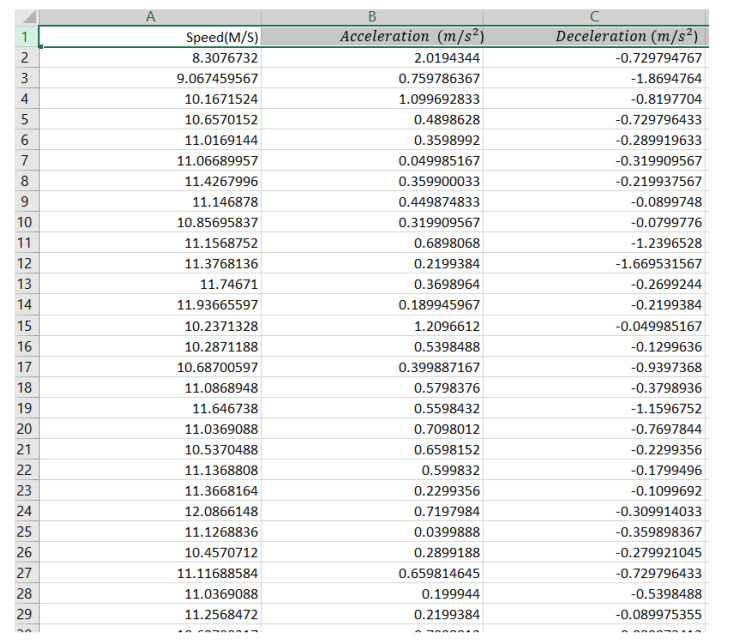
Example results of acceleration and deceleration.

One of this study’s goals was to predict driving style depending on the analysis of the driver’s activity via acceleration and deceleration (braking), which describe the motion of a motorcycle. Therefore, these parameters are fundamental to defining the behavior of driving. The acceleration and deceleration are calculated based on the differences in motorcycle speed per second. After that, each one of the data types was exported to an Excel sheet (Figure 4) and placed in its table with the key field “deceleration and acceleration”, which corresponds to the event’s time.

**Figure 5:**
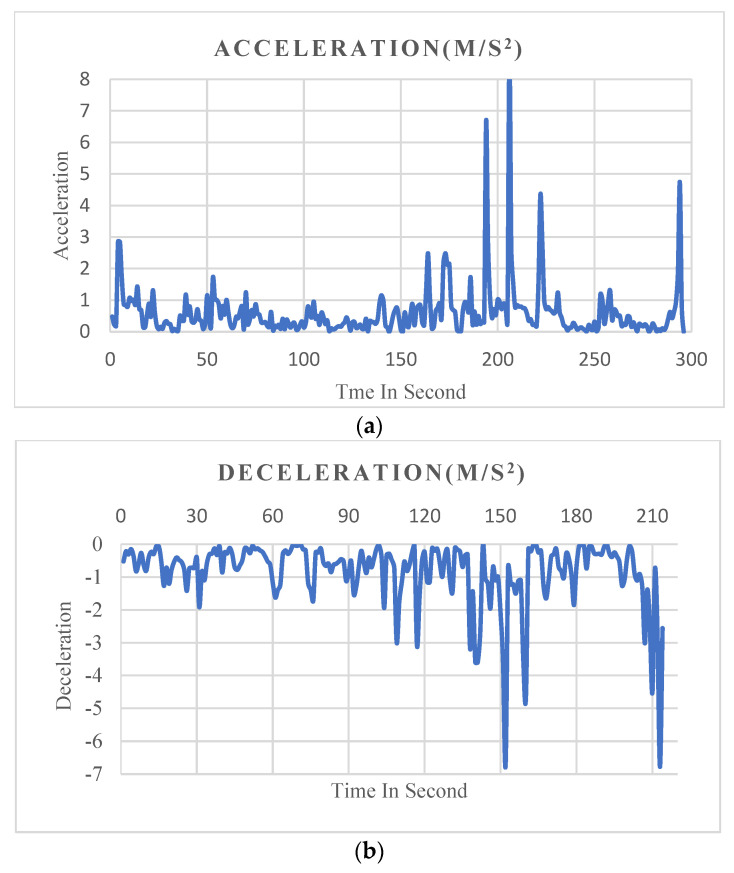
Graphs representing acceleration and deceleration. (**a**) Acceleration. (**b**) Deceleration (braking).

The acceleration and deceleration are calculated by meters for each second squared (m/s^2^). Figure 5 shows an example of graphs representing acceleration and deceleration (braking).

### 3.3. Data Cleaning

**Figure 6:**
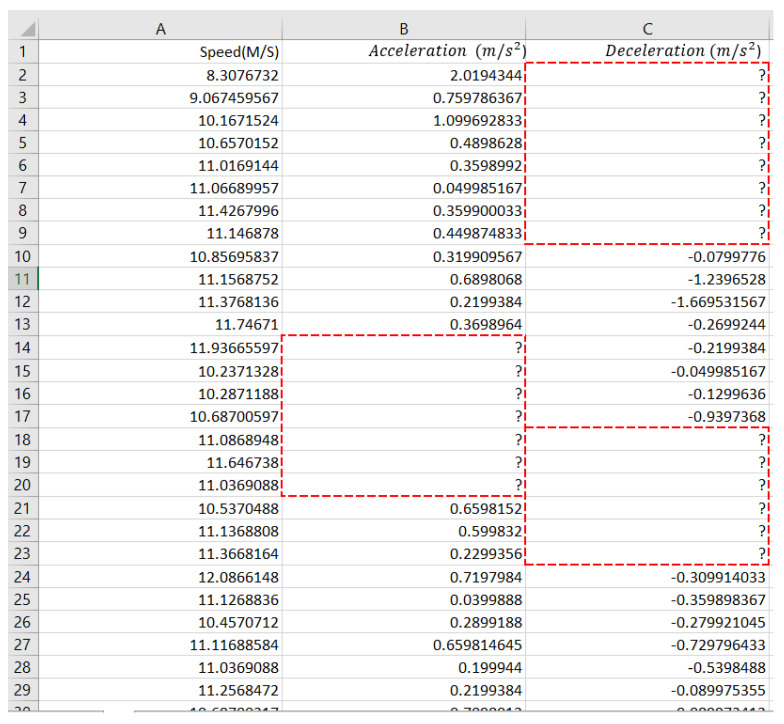
Sample of fields that need cleaning.

In the dataset, all attributes must be numerical. However, some rows were with no data. Moreover, some columns were not in numerical format. Figure 6 shows some columns and row formats for data cleaning.

### 3.4. Measuring the Driving Profile

The focus of this study is on driving behavior on various roadways. The driving profile chosen for analysis was one in which the driver drove from a starting point until the motorcycle came to a complete halt at the end stop. The driver might have experienced varying levels of acceleration and braking aggressiveness during this time. The most important factors that will be relied upon during the data collection process for the various types of devices used are the speed profile and the acceleration/deceleration process.

#### 3.4.1. Driver Profiling Rules

Identifying the driving behavior of drivers could facilitate more effective traffic safety work and allow measures to be tailored for a specific driver group. It is possible to detect safety-critical driving behavior by studying fluctuations in the acceleration and deceleration profiles. Threshold values can be used to separate aggressive and dangerous from normal driving behavior. For motorcycle driver behavior, a threshold issue is raised. For instance, car driver behavior entails several thresholds, and events can be classified as normal acceleration (1.5 to 3.5 m/s^2^), aggressive acceleration (3.5 to 7 m/s^2^), and dangerous acceleration (7 to 12 m/s^2^). As for the deceleration event, it can be classified into normal deceleration (−3 to −5.5 m/s^2^), aggressive deceleration (−5.5 to −9 m/s^2^), and dangerous deceleration (−9 to −14 m/s^2^) [39,40].

However, motorcycles do not have special thresholds when classifying driver behavior events [41,42]. Hence, a follow-up question is raised: How should thresholds be obtained to determine motorcycle driver behavior? The other question related to the validity and generality of these mentioned thresholds concerning communities can be elaborated as follows: If thresholds apply to US samples, are they also valid for samples in other countries? One of the suggestions is to extract the event from a sample of drivers in each country and investigate the distribution of these events for a particular sample. The most frequent event can be utilized as the normal sample, whereas the less frequent event can be classified as aggressive [43]. Therefore, the standard deviation hypotheses were adopted to obtain the threshold for speed and acceleration for motorcycles through the case study, and the assumption that was adopted to calculate the driving thresholds for motorcycles showed results that are close to the values of the driving thresholds for cars mentioned in the previous works.

#### 3.4.2. Threshold Formulation for Behavior Classification

**Figure 7:**
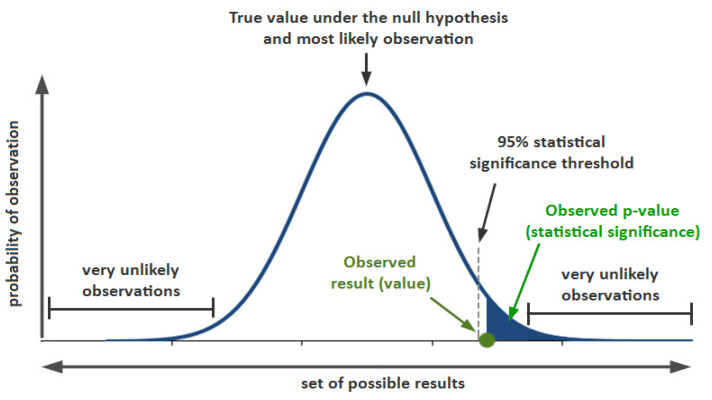
A plot of normal distribution [45].

Several researchers addressed the threshold of acceleration/deceleration limits of vehicles that are associated with causalities and a high risk of being involved in an accident. However, they did not specify the context for estimating it [39,40]. On the other hand, there are studies in other domains that extracted the thresholds using the standard deviation, such as [44], in which the calculation of (mean ± 2 standard deviations (sdev)) to estimate threshold values was performed by dividing background data from anomalies. The exact value of (mean + 2 sdev) is still used by some researchers as the threshold [44], where extracting the thresholds to identify the acceleration/deceleration limits of motorcycles has been using the calculation of (mean ± 2 standard deviations (sdev)) by assuming the first standard deviation is the most likely event to be observed. Since a group of drivers has been randomly selected to analyze behavior and determine an event, it was assumed that the most observed behavior is to be labeled as normal. Otherwise, the behavior is labeled as abnormal and relies on a positive direction (increase acceleration and increase deceleration to assume abnormality). Figure 7 shows a plot of normal distribution.

The plot of normal distribution above consists of two parts, with the first part consisting of a negative value (value < average). These values have little effect on traffic accidents. However, this effect was not due to behavior but rather due to slow speed that causes a delay in the training. The second part of the distribution represents the positive value (average < value), consisting of the most frequent events that affect the traffic accidents, so the positive part of the distribution is used to determine the threshold of the driving behavior of the motorcyclist. One standard deviation of data encompasses approximately 68.2% of outcomes in the distribution of occurrences based on current behavior. Two standard deviations of data encompass approximately 95.4% of outcomes in the distribution of occurrences based on current behavior. Three standard deviations of data encompass approximately 99.7% of outcomes in the distribution of occurrences based on current behavior. In mathematical notation, these facts can be expressed as follows:

Pr (μ − 1 σ ≤ X ≤ μ + 1 σ) ≈ 68.27%(1)

Pr (μ − 2 σ ≤ X ≤ μ + 2 σ) ≈ 95.45%(2)

Pr (μ − 3 σ ≤ X ≤ μ + 3 σ) ≈ 99.7%(3)

where

Pr () is the probability function;
Χ is an observation from a normally distributed random variable;
μ (mu) is the mean of the distribution;
σ (sigma) is its standard deviation.

##### Threshold of Acceleration Limits

The threshold was set for acceleration as follows:

**Step 1**: Calculate average acceleration (m/s^2^) by an Equation (4).
  (4) $$\mathrm{average}\;\mathrm{of}\;\mathrm{acceleration}\;(m/s^{2})=\frac{\sum_{i=1}^{n}Ai\;}{n\;}.\;$$
  where
  Ai *=* average of acceleration during a certain period;
  *n =* number of accelerations.
**Step 2**: Define the normal acceleration threshold, which has been formulated by Equation (5) and Equation (1) to be Equation (6).
  (5) $$\mathrm{Standard}\;\mathrm{Deviation}\;\sigma =\sqrt{\frac{1}{N-1}\sum_{i=1}^{N}X_{i}-\bar{X}}\;$$
  where
  *N =* number of accelerations during a period;
  *X_i_ =* value of acceleration;
  $\bar{X}$*=* average of acceleration (m/s^2^).
    (6) $$\mathrm{Normal}\;\mathrm{accelaration}(m/s^{2})\leq \mathrm{Average}\;\mathrm{accelaration}+\mathrm{Standard}\;\mathrm{Deviation}$$
    where
    Average acceleration = mean
**Step 3**: Define the aggressive acceleration threshold by assuming the less frequent event of acceleration is aggressive acceleration, by formulating Equation (7) from Equations (1) and (2).
  (7) (average acceleration+standard devaition)<aggressive acceleration≤(average acceleration+(2∗standard devaition))
  where
  Average acceleration = mean
**Step 4**: Define dangerous acceleration, which has been formulated from Equation (8) by assuming the least frequent events are dangerous acceleration.
  (8) dangerous dcceleration>(average of acceleration+(2∗standard Deviation) )
**Step 5**: The results of thresholds for acceleration are:
  - Normal acceleration (acceleration ≤ 1.3)
  - Aggressive acceleration (1.3 < acceleration and acceleration ≤ 2.5)
  - Dangerous acceleration (acceleration > 2.5)

**Figure 8:**
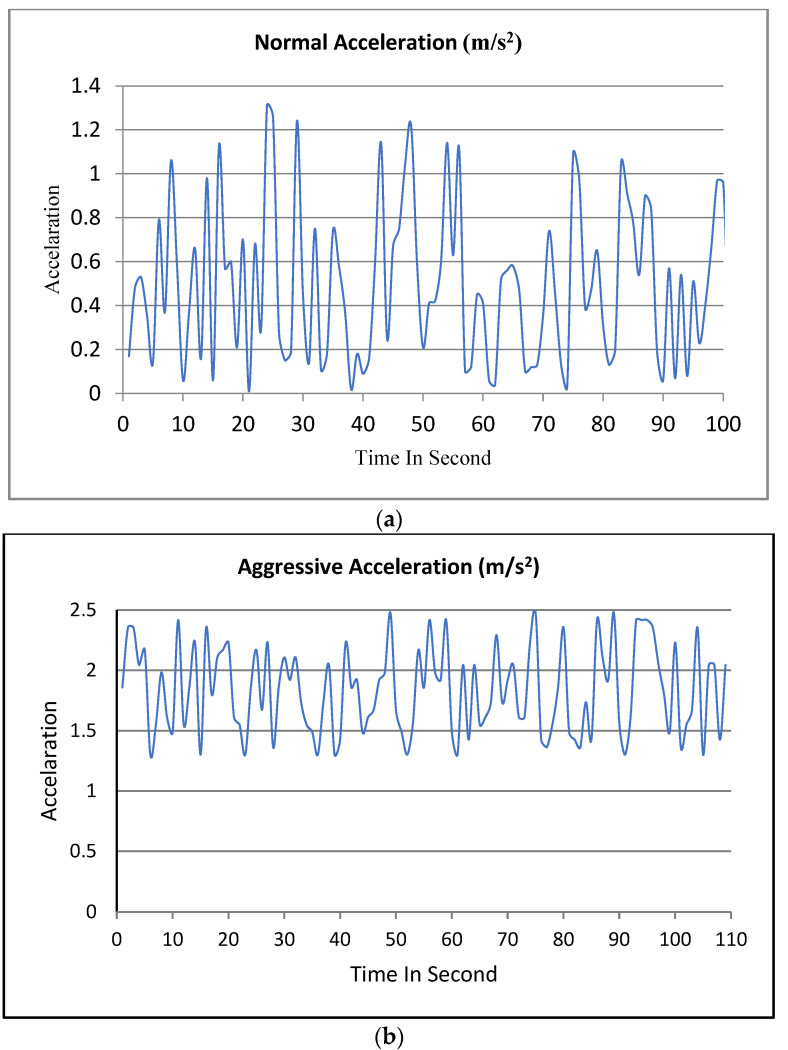

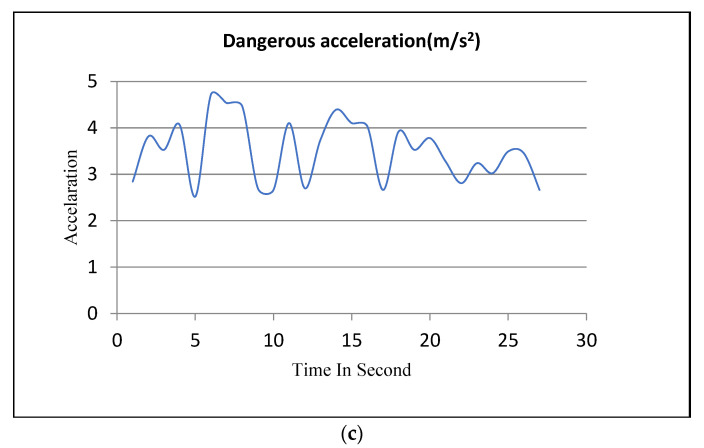
An example of normal, aggressive, and dangerous acceleration. (**a**) Normal acceleration. (**b**) Aggressive acceleration. (**c**) Dangerous acceleration.

Figure 8 below presents an example of normal, aggressive, and dangerous acceleration after collecting the limits of acceleration. The deceleration limits were calculated by identifying the threshold in the next section.

##### Threshold of Deceleration (Braking) Limits

The threshold was set for deceleration as follows:

**Step 1**: Calculate average deceleration (m/s^2^) with Equation (9).
  (9) $$\mathrm{average}\;\mathrm{of}\;\mathrm{deceleration}\;(m/s^{2})=\frac{\sum_{i=1}^{n}Ai\;}{n\;}$$
  where
  Ai *=*Deceleration for driving during a certain period
**Step 2**: Define the normal deceleration threshold with Equation (10), then from Equation (1), which has been formulated in Equation (11).
  (10) $$\mathrm{Standard}\;\mathrm{Deviation}\;\sigma =\sqrt{\frac{1}{N-1}\sum_{i=1}^{N}X_{i}-\bar{X}}\;$$
  where
  *N* = number of decelerations during a period;
  *X_i_* = value of the deceleration;
  $\bar{X}$ = average of acceleration (m/s^2^).
    (11) $$\mathrm{normal}\;\mathrm{deceleration}\;(m/s^{2})\leq \mathrm{average}\;\mathrm{deceleration}+\mathrm{Standard}\;\mathrm{deviation}\;$$
    where average deceleration = mean.
**Step 3**: Define the aggressive deceleration threshold by assuming the least frequent events of deceleration are aggressive deceleration, where Equations (1) and (2) have been formulated in Equation (12).
  (12) (average deceleration+standard devaition) < aggressive deceleration≤ (average Deceleration+(2∗standard deviations))
  where average deceleration = mean.
**Step 4**: Define the dangerous deceleration from Equation (2) by assuming the least frequent events are dangerous decelerations with Equation (13).
  (13) dangerous deceleration>(average of deceleration+(2∗Standard Deviation))
**Step 5**: The results of thresholds for acceleration are:
  - Normal deceleration (deceleration ≥ −9.54)
  - Aggressive deceleration (−6.01 < aggressive deceleration ≥ −9.54)
  - Dangerous deceleration (deceleration < −6.01)

**Figure 9:**
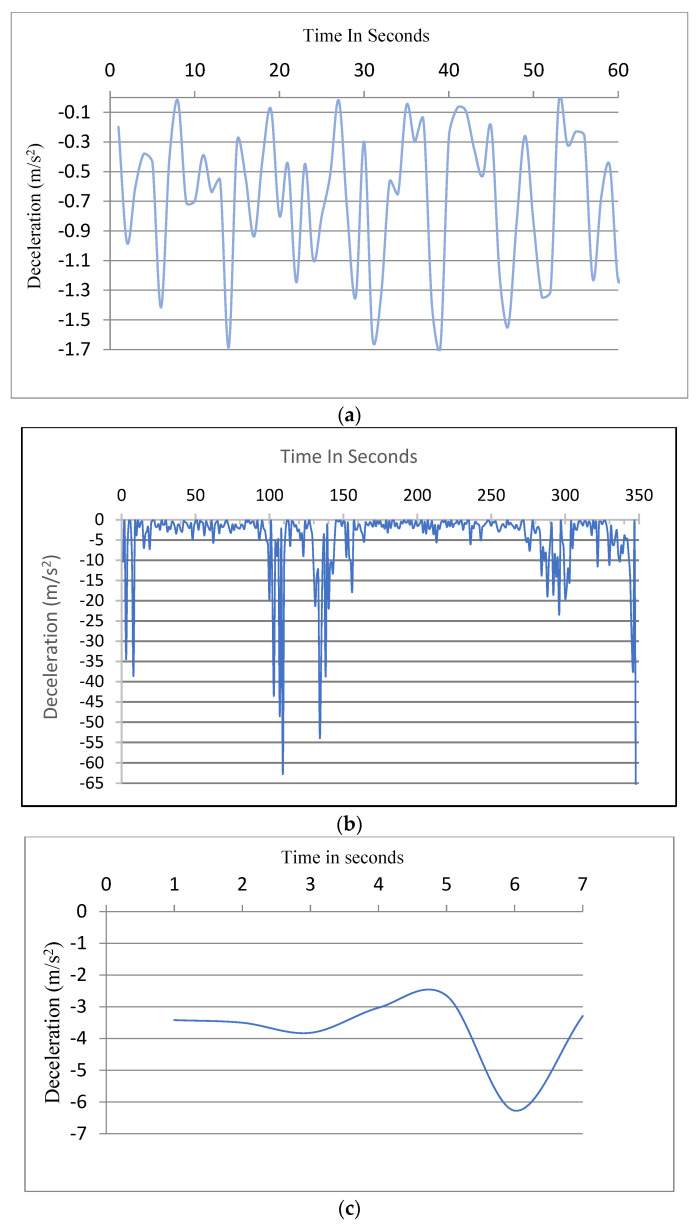
An example of normal, aggressive, and dangerous deceleration. (**a**) Normal deceleration. (**b**) Aggressive deceleration. (**c**) Dangerous deceleration.

Figure 9 below presents examples of normal, aggressive, and dangerous deceleration.

Note that the values of acceleration/deceleration limits of motorcycles obtained in our study are closer to the acceleration/deceleration limits of cars, so assume that the previous studies used the standard deviation method to calculate the acceleration/deceleration limits of cars. On the other hand, the values of acceleration/deceleration limits of motorcycles are closer but do not have similar values to the thresholds of cars because the dataset in our study collects from motorcycles, while other studies collected datasets from vehicles.

### 3.5. Driving Behavior Analysis

The focus of this study is driving behavior on a short road distance on a highway and in-city road. The driving profile chosen for analysis was the driver’s behavior while driving during that time; the driver might have encountered varying levels of acceleration and braking aggression. To assess the event and the effect of driving behavior in Table 1, a simple program utilizing RapidMiner is constructed.

After dividing the occurrence of each event by the total of events (including the zero-acceleration event), we obtained the ratio of each event per second, reported in Table 2.

To summarize the analysis of the results, Table 2 above shows that motorcycle drivers completed the trip at 3321.3 km/h, and the values of acceleration for aggressive and dangerous driving events are 100.68 and 82.56, respectively, compared to the values of deceleration for aggressive events at 89.25 and dangerous events at 80.06. In general, drivers produce a slightly higher number of aggressive and dangerous events for acceleration. Table 3 presents normal, aggressive, and dangerous events.

The last feature discussed in this section is the speed of the drivers. Based on the selected road, the maximum speed is 80 km/h. Table 4 shows the occurrence of over-speed behavior for motorcycle drivers.

According to Table 4 above, the ratio of over-speed on average is 19% of the total events compared to 79% of regular speed events. Therefore, this ratio is considered high, and three subjects were observed with more than 50% over-speed events.

## 4. Discussion of Results

The calculation of dangerous and aggressive behavior performance with the use of a percent of dangerous and aggressive values assessed based on the various driving modes of the motorcycle was defined as a key concept for this research. To achieve our research objectives, an experiment was conducted in Malaysia from mid-July to mid-September 2020, taking place in Tanjung Malim and Selangor.

In the experiment, 16 drivers were selected randomly from local Malaysian students aged 18–35 years old from UPSI and UNITEN, and the drivers drove via different paths in Tanjung Malim and Selangor on two different roads types (urban (in-city) and highway). The data were analyzed to calculate the normal, aggressive, and dangerous acceleration and deceleration events for each driver, and most of the results achieved were between average accelerations (1678) and normal decelerations (1284).

**Figure 10:**
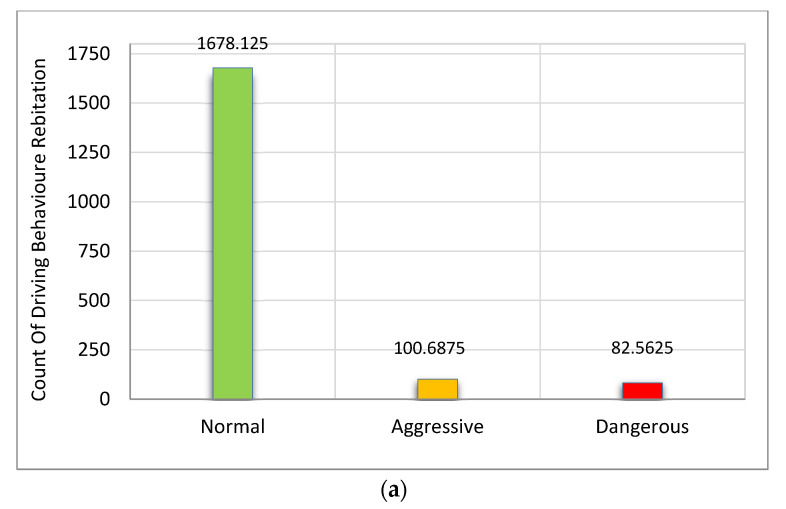

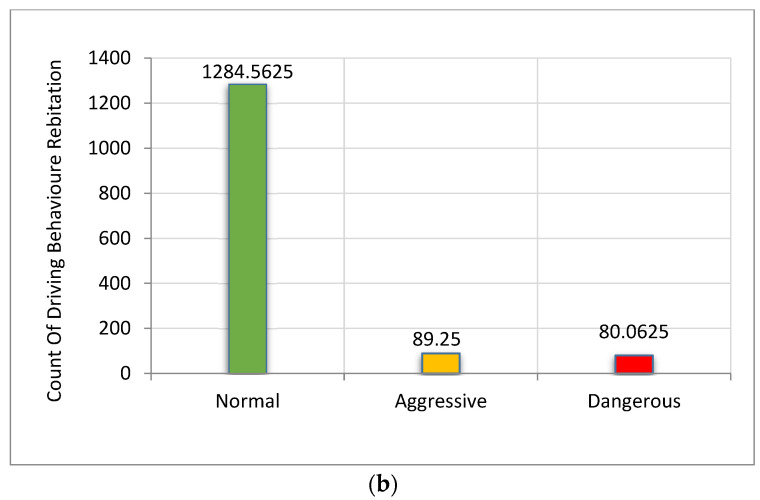
Normal driving (89% total of everyday driving). (**a**) Type of driving behavior in case of accelerations. (**b**) Type of driver behavior in case of decelerations.

Moreover, the results indicate that a change in driving behavior toward the aggressive and dangerous was few: 182 in case of accelerations and 169 in case of decelerations out of 53141 readings. Therefore, it is concluded that 88% of the readings were recorded under the normal situation, as shown in Figure 10.

**Figure 11:**
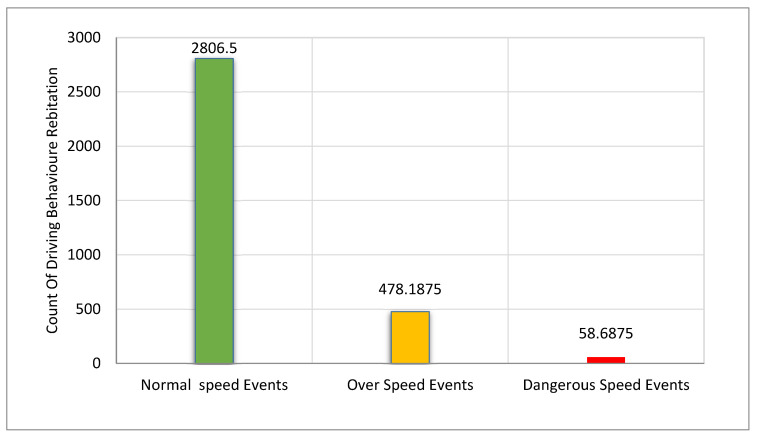
Type of driver behavior concerning speed.

These event percentages increase with the increase in the distance and can negatively affect the safety of the road and the driver, causing an increase in accidents because the percentage increase was achieved within a short period. The longer the driving period, the higher the risk of accidents. This reconfirms the previous studies which mentioned that drivers’ behaviors cause 40% of accidents. The last feature discussed in this section is the speed of the drivers. Based on the selected road, the maximum speed is 80 km/h. Most of the results indicate a change in driving behavior toward the over-speed and dangerous speed with 478.1875 and 58.6875 readings out of 3343.375 readings as shown in Figure 11. Therefore, over 22% of motorcycle riders exceed the speed limit in all road categories, and their speed is often higher compared to that of cars. Speeding is also a major contributor to all road crashes, with speed being a factor in 34% of all fatal motorcycle accidents.

## 5. Conclusions

Despite the growing interest in safety research, the current studies in this field have some disadvantages. According to previous studies, further development and research are required to solve such constraints, particularly in the area of driving behavior. The recognition of aggressive driving is a crucial step in taking steps to improve the problem. This research intends to evaluate motorcycle driving behavior and detect aggressive behavior as a measure to improve road safety and prevent accidents. The technique proposed in this study attempts to understand drivers’ conduct throughout day-to-day trips by evaluating their moving activity data recorded through the Speedometer GPS application. This application, based on the Android operating system, is supplemented with spatiotemporal data, which includes the speed of drivers while driving, and is used for identifying and evaluating driving behavior. We suggest a stratification of acceleration/deceleration and speed consisting of various classes to test driving speed and braking based on our experiments. A severity stratification of acceleration/deceleration presented the following parameters: normal acceleration (acceleration (m/s^2^) <= 4.73), aggressive acceleration (4.73 < acceleration (m/s^2^) and acceleration (m/s^2^) <= 7.48), dangerous acceleration (acceleration > 7.48), normal deceleration (deceleration (m/s2) => -6.01), aggressive deceleration (deceleration (m/s^2^) < −6.01 and deceleration (m/s^2^) => −9.54), and dangerous deceleration (deceleration (m/s^2^) < −9.54). The results confirm what was mentioned in previous studies, which is that driving behavior causes 40% of all serious and fatal crashes, and aggressive driving style is specified as a behavioral pattern or categorization of a driver which is associated with the profiles of dangerous speeding and inconsistent or excessive acceleration and deceleration with over 50% of motorcyclists riding above the speed limit stipulated at all road categories, and sometimes even higher. In future research, we want to use the collected datasets as input values in a machine learning environment to estimate parameter values in the future using prediction algorithms, decision trees, or time series analysis, and compare the outcomes. There is the possibility of developing an analytical model based on the behavioral factors and, in addition, steering wheel angle movement including toll (degrees), pitch (degrees), and yaw (degrees) as a future study.

## Figures and Tables

**Table 1:** Driver profiling.

Drivers	Accelerations			Decelerations			Zero	Total
	Normal	Aggressive	Dangerous	Normal	Aggressive	Dangerous		
D 1	1393	90	50	1108	60	56	5	2762
D 2	1827	168	150	1388	126	163	8	3830
D 3	4220	305	218	3154	279	219	6	8401
D 4	4089	183	143	3050	180	120	10	7775
D 5	497	42	25	346	42	29	2	983
D 6	1285	39	40	972	40	30	6	2412
D 7	757	44	20	520	40	26	3	1410
D 8	1466	154	114	966	116	129	3	2948
D 9	1339	28	29	1018	30	26	6	2476
D 10	1463	102	53	1043	79	65	4	2809
D 11	907	50	81	656	56	63	5	1818
D 12	684	60	51	530	66	48	3	1442
D 13	1387	91	125	1134	93	94	6	2930
D 14	1458	70	73	1174	61	65	8	2909
D 15	3121	137	98	2787	126	94	10	6373
D16	957	48	51	707	34	54	12	1863
**Average**	**1678.12**	**100.68**	**82.56**	**1284.5**	**89.25**	**80.06**	**6.06**	**3321.3**

**Table 2:** Acceleration and deceleration event ratios for drivers.

Drivers	Acceleration Decelerations						Zero
	Normal	Aggressive	Dangerous	Normal	Aggressive	Dangerous	
D 1	0.50434467	0.0325850	0.0181028	0.40115858	0.0217233	0.0202751	0.001810
D 2	0.47702349	0.0438642	0.0391644	0.36240208	0.0328981	0.0425587	0.002088
D 3	0.50232112	0.0363052	0.0259492	0.37543149	0.0332103	0.0260683	0.000714
D 4	0.52591639	0.0235369	0.0183922	0.39228295	0.0231511	0.0154340	0.001286
D 5	0.50559517	0.0427263	0.0254323	0.35198372	0.0427263	0.0295015	0.002034
D 6	0.53275290	0.0161691	0.0165837	0.40298507	0.0165837	0.0124378	0.002487
D 7	0.53687943	0.0312056	0.0141849	0.36879432	0.0283687	0.0184397	0.002127
D 8	0.49728629	0.0522388	0.0386702	0.32767978	0.0393487	0.0437584	0.001017
D 9	0.54079159	0.0113085	0.0117124	0.41114701	0.0121163	0.0105008	0.002423
D 10	0.52082591	0.0363118	0.0188679	0.37130651	0.0281238	0.0231399	0.001423
D 11	0.49889989	0.0275027	0.0445544	0.36083608	0.0308030	0.0346534	0.002750
D 12	0.47434119	0.0416088	0.0353675	0.36754507	0.0457697	0.0332871	0.002080
D 13	0.47337884	0.0310580	0.0426621	0.38703071	0.0317406	0.0320819	0.002047
D 14	0.50120316	0.0240632	0.0250945	0.40357511	0.0209694	0.0223444	0.002750
D 15	0.48972226	0.0214969	0.0153773	0.43731366	0.0197709	0.0147497	0.001569
D16	0.51368760	0.0257648	0.0273752	0.37949543	0.0182501	0.0289855	0.006441
**Average**	**0.50525959**	**0.0303155**	**0.0248583**	**0.38676351**	**0.0268719**	**0.0241056**	**0.001825**

**Table 3:** Normal, aggressive, and dangerous events.

*Features*	*Samples*
*Sample size*	16
*Number of trips*	116
*Total driving distance of the sample*	1240 k/h
*Normal accelerations*	1678.125
*Aggressive accelerations*	100.6875
*Dangerous accelerations*	82.5625
*Normal deceleration*	1284.5625
*Aggressive deceleration*	89.25
*Dangerous deceleration*	80.0625
*Zero events*	6.0625
***Total***	**3321.3**

**Table 4:** The occurrence of over-speed behavior for the drivers.

*Drivers*	*Normal Speed Events*	*Over-Speed Events*	*Dangerous Speed Events*	*Normal Speed Ratio*	*Over-Speed Ratio*	*Dangerous Speed Ratio*
D 1	2094	699	0	75%	25%	0
D 2	2961	831	38	77%	22%	1%
D 3	8470	0	0	100%	0	0
D 4	7741	143	0	98%	2%	0
D 5	998	0	0	100%	0	0
D 6	849	1128	468	35%	46%	19%
D 7	1434	2	0	99.9%	0.1%	0
D 8	2458	516	0	83%	17%	0
D 9	746	1717	13	30%	69%	1%
D 10	2277	363	198	80%	13%	7%
D 11	1056	667	97	58%	37%	5%
D 12	1454	0	0	100%	0	0
D 14	2520	368	19	87%	13%	1%
D 15	6344	40	0	99%	1%	0
D16	1057	696	102	57%	38%	5%
**Average**	**2806.5**	**478.1875**	**58.6875**	**79%**	**19%**	**2.4%**

## Data Availability

Not applicable.
